# Electroacupuncture Improved Hippocampal Neurogenesis following Traumatic Brain Injury in Mice through Inhibition of TLR4 Signaling Pathway

**DOI:** 10.1155/2017/5841814

**Published:** 2017-08-07

**Authors:** Yuqin Ye, Yongxiang Yang, Chen Chen, Ze Li, Yanfeng Jia, Xinhong Su, Chaoxian Wang, Xiaosheng He

**Affiliations:** ^1^Department of Neurosurgery, Xijing Hospital, Fourth Military Medical University, Xi'an 710032, China; ^2^Department of Neurosurgery, PLA 163rd Hospital (Second Affiliated Hospital of Hunan Normal University), Changsha 410000, China; ^3^Department of Neurology, PLA 422nd Hospital, Zhanjiang 524005, China; ^4^Institute of Psychology, Fourth Military Medical University, Xi'an 710032, China; ^5^Department of Neurosurgery, The Fourth People's Hospital of Shaanxi Province, Xi'an 710034, China

## Abstract

The protective role of electroacupuncture (EA) treatment in diverse neurological diseases such as ischemic stroke is well acknowledged. However, whether and how EA act on hippocampal neurogenesis following traumatic brain injury (TBI) remains poorly understood. This study aims to investigate the effect of EA on hippocampal neurogenesis and neurological functions, as well as its underlying association with toll-like receptor 4 (TLR4) signaling in TBI mice. BrdU/NeuN immunofluorescence was performed to label newborn neurons in the hippocampus after EA treatment. Water maze test and neurological severity score were used to evaluate neurological function posttrauma. The hippocampal level of TLR4 and downstream molecules and inflammatory cytokines were, respectively, detected by Western blot and enzyme-linked immunosorbent assay. EA enhanced hippocampal neurogenesis and inhibited TLR4 expression at 21, 28, and 35 days after TBI, but the beneficial effects of EA on posttraumatic neurogenesis and neurological functions were attenuated by lipopolysaccharide-induced TLR4 activation. In addition, EA exerted an inhibitory effect on both TLR4/Myd88/NF-*κ*B and TLR4/TRIF/NF-*κ*B pathways, as well as the inflammatory cytokine expression in the hippocampus following TBI. In conclusion, EA promoted hippocampal neurogenesis and neurological recovery through inhibition of TLR4 signaling pathway posttrauma, which may be a potential approach to improve the outcome of TBI.

## 1. Introduction

As one of the leading life-threatening diseases worldwide, traumatic brain injury (TBI) often causes high mortality or a range of severe neurological deficits and long-term disability due to an inadequate self-repair capacity of the central nervous system (CNS) [1]. For a long time, neural stem cells (NSCs) have been identified in the subgranular zone (SGZ) of hippocampal dentate gyrus (DG), which contributed to endogenous neurogenesis throughout life [2, 3]. The remarkable characteristics of hippocampal NSCs include self-renew, production of newborn neurons, and integration into the damaged neural network following CNS injury, which makes intervention of endogenous neurogenesis as an attracting strategy for the rehabilitation of injured brain. However, accumulating evidence indicated that posttraumatic neurogenesis in the hippocampus was insufficient to overcome the neural damage caused by TBI [4, 5]. Therefore, it is required to discover an approach to enhance hippocampal neurogenesis for brain reconstruction and rehabilitation after TBI.

Acupuncture, an ancient curing skill, has been applied in relieving pain and boosting body energy since about 2500 BC in China [6]. Electroacupuncture (EA), originating from traditional acupuncture around the 1930s, has been verified to significantly improve the therapeutic effects of the traditional acupuncture in a variety of diseases [7, 8]. In recent years, increasing studies have indicated that EA was conducive to the improvement of neurological function following CNS damage via stimulation of certain acupuncture points, whereas the detailed mechanism was still not fully understood [8–10]. Furthermore, a lot of researches up to now have emphasized the neuroprotection of EA in brain stroke such as cerebral ischemia and intracerebral hemorrhage, but few studies focused on the role of EA in brain trauma, especially concerning the effect of EA on posttraumatic neurogenesis and its potential linkage with the pathophysiological process in the hippocampus after TBI [11–13]. Hence, a better understanding of EA in traumatic brain might provide new clues to promote the inadequate neurogenesis in the hippocampus following TBI.

Previous studies have demonstrated that the therapeutic mechanisms of EA in neurological diseases involved a series of molecules and pathophysiological processes, such as angiopoietin 1- and 2-mediated angiogenesis, mammalian target of rapamycin-associated neuronal autophagy, and *α*7 nicotinic acetylcholine receptor-related inflammatory responses [9, 12, 14, 15]. As an important member of pattern-recognition receptor family, toll-like receptor 4 (TLR4) has been found in diverse cell types including microglia, astrocyte, and neuron in the CNS [16]. A growing body of evidence suggested that TLR4 signaling pathway played a key role in inflammation of CNS diseases, such as cerebral stroke, Alzheimer's disease, and spinal cord injury [17–20]. Therefore, it has been proposed as a therapeutic target. TLR4 recognized not only pathogen-associated molecular patterns (PAMP) but also damage-associated molecular patterns (DAMP), which could induce intracellular cascade activation and release inflammatory cytokines in response to the pathological condition of injured brain [19, 21, 22]. In addition, several groups have provided evidence to support the crucial role of TLR4 signaling pathway in governing NSC proliferation and differentiation [23–26]. Our previous study also revealed that the expression of hippocampal TLR4 increased significantly and varied in a similar temporal pattern to posttraumatic NSC proliferation in SGZ [27]. However, whether there is any involvement of TLR4 pathway in the mechanism by which EA works in hippocampal neurogenesis after TBI remains unclear.

Therefore, the current study was performed to investigate the effect of EA treatment on neurogenesis in the hippocampus of experimental TBI mice. Moreover, the involvement of TLR4 and its downstream cascade in the potential mechanism of EA-related neurogenesis were also explored. The finding might be helpful for novel insight into neural reparation and functional recovery after TBI.

## 2. Materials and Methods

### 2.1. Experimental Design

Male C57BL/6 mice, weighing 18–20 g, were provided by the Experimental Animal Center of Fourth Military Medical University (Xi'an, China). Animals were housed in a standard laboratory bedding environment (22.0 ± 2°C) and maintained on a controlled 12-hour light-dark cycle (light on 08:00–20:00). Enough food and water were available for all animals. The present experimental protocols and animal procedures complied with the National Experimental Animal Guidelines and were approved by the Fourth Military Medical University Ethic Committee (FMMUEC). All efforts were taken to minimize animal suffering throughout the experimental duration.

The first experiment was designed to investigate the effect of EA treatment on hippocampal neurogenesis and TLR4 expression after TBI. Seventy-two mice were randomly divided into four groups: sham, sham + EA, TBI, and TBI + EA groups (*n* = 18 in each). The sham group received sham injury operation; the TBI group was subjected to TBI treatment; the TBI + EA group was treated with EA postinjury. Immunofluorescence (IF) staining, water maze test (WMT), and neurological severity score (NSS) test were performed to evaluate the neurogenesis, neurocognitive, and neurobehavioral functions at 21, 28, and 35 days after TBI. The protein and mRNA level of TLR4 were, respectively, detected by Western blot (WB) and real-time PCR.

In the second experiment, TLR4 ligand lipopolysaccharide (LPS) was used to activate TLR4 in the hippocampus. The effects of TLR4 activation on EA-related neurogenesis, neurocognitive, and neurobehavioral functions following TBI were explored. Twenty-seven mice were randomly divided into three groups: TBI + EA, TBI + EA + LPS, and TBI + EA + vehicle (Veh) groups (*n* = 9 in each). The TBI + EA group underwent the same treatment as above; the TBI + EA + LPS group was subjected to EA treatment and LPS administration posttrauma; the TBI + EA + Veh group received EA treatment and vehicle endotoxin-free water (solvent of LPS) injection posttrauma. The neurogenesis, neurocognitive, and neurobehavioral functions were, respectively, assessed by IF staining, WMT, and NSS test as described above.

In the third experiment, downstream molecules and inflammatory cytokines of TLR4 pathway were determined to further disclose the potential mechanism of EA-related neurogenesis in the hippocampus posttrauma. Thirty mice were randomly divided into six groups: sham, sham + EA, TBI, TBI + EA, TBI + EA + LPS, and TBI + EA + Veh groups (*n* = 6 in each). Each group was subjected to the same treatment as above, respectively. The expression of downstream molecules in TLR4 pathway was examined with WB, and the level of inflammatory cytokines was detected by enzyme-linked immunosorbent assay (ELISA) at 35 days after TBI.

### 2.2. Establishment of TBI Mouse Model

Following intraperitoneal (i.p.) chloralhydrate (400 mg/kg) anesthesia, controlled cortex injury (CCI) was produced in mice to establish TBI model. The mice were secured in a stereotaxic frame (Kopf Instruments, Tujunga, CA, USA) by an incisor bar and two lateral ear pins. An incision was made at the midline on the scalp, and the fascia was reflected to expose the skull for craniotomy. The drilling site was between the lambda and bregma and 2.5 mm lateral to the sagittal suture in the right hemisphere. After the skull flap (4.0 mm diameter) was removed, brain contusion was produced on the exposed dura using a CCI device (Hatteras Instruments, Cary, NC, USA). According to our previous study [28], the impact parameters were set at 1.0 mm for cortical impact depth, 3.0 m/s for impact velocity, and 100.0 ms for contact time. Briefly, a piston rod with an impact tip of 3.0 mm diameter was centered at craniotomy site and impacted dura perpendicularly to contuse the underlying cortex. Then, the skull flap was reset, the scalp was sutured with nylon threads, and incision was cleaned with sterile alcohol. The mice in the control group were treated only with craniotomy but not cortical impact. Animal core temperature was maintained at 37.0 ± 0.5°C with a heating pad during surgical operation and postsurgical recovery period.

### 2.3. Electroacupuncture Treatment

After animals were anesthetized, ST36 acupoint (“Zusanli”, locating at 5.0 mm distal to the head of the fibula under the knee joint and 2.0 mm lateral to the tubercle of the anterior tibia) and GV40 acupoint (“Dazhui”, locating at the posterior midline and the depression below the spinous process of the seventh cervical vertebra) were selected for EA. Each of two stainless steel needles of 0.3 mm diameter was inserted at a depth of 3.0 mm into the acupoints, respectively, with its end connecting to the output terminal of an EA instrument (Model SDZ-V, SMACL, Suzhou, China). The stimulation parameters were modified from previous studies taken by the Anesthesiology Department of our hospital [29, 30]. EA treatment started at the next day after TBI and continued for 35 consecutive days in accordance with the parameters: alternating dense-sparse wave; 2/15 Hz for frequency; 1.0 mA for current intensity; 30 min per day. Mouse body temperature was maintained at 37.0 ± 0.5°C by a heating pad during EA treatment.

### 2.4. Drug Administration

Thymidine analog bromodeoxyuridine (BrdU) (Sigma-Aldrich, B9285, St. Louis, MO, USA) was used to label endogenous NSCs in SGZ for neurogenesis evaluation. BrdU was dissolved in sterile saline solution to a concentration of 10.0 mg/ml before i.p. injection. The mice received a pulse of BrdU (100 mg/kg) injection once per day at 1–7 days posttrauma and were sacrificed at 28 days after the last injection (namely, at the 35th day following TBI).

Evidence showed that ultrapure LPS from *E. coli* serotype 0111:B4 (Invivogen, tlrl-3pelps, San Diego, CA, USA) was extracted with special steps and only activated TLR4 signaling pathway [31]. At 30 minutes before onset of EA, LPS was dissolved in endotoxin-free water to a final concentration of 0.5 mg/ml and was injected into the right lateral ventricle with a dosage of 250 *μ*g/kg using a microliter syringe (Hamilton, Reno, NV, USA) at the coordinates: 2.0 mm posterior to the bregma, 1.5 mm lateral to midsagittal line, and 2.5 mm ventral from the skull surface [32]. For mice in the Veh group, identical volume injection of endotoxin-free water was given at the same coordinates.

### 2.5. Immunofluorescence Staining

Mouse was anesthetized and perfused intracardially with 4% paraformaldehyde in 0.1 M phosphate-buffered saline (PBS) for 1 hour. The brain tissue was removed and immersed in 4% paraformaldehyde at 4°C overnight and then dehydrated by alcohol and embedded in paraffin. Next, 5 *μ*m thick coronal sections from −1.50 to −3.56 mm of the bregma (covering the DG of the hippocampus) were prepared in a microtome (Leica, Nussloch, Germany) and dried at 94°C overnight. Ten sections (100 *μ*m apart) from each mouse brain were selected and deparaffinized by alcohol and dimethylbenzene. For DNA denaturation, the sections were incubated in citric acid antigen retrieval buffer (pH = 6.0) at 95°C for 10 min. To block nonspecific signals, the sections were then incubated in PBS with 1% donkey serum albumin and 0.3% Triton X-100 at room temperature for 30 min.

Newly generated neurons in the hippocampus were double-labeled by BrdU/NeuN to assess the neurogenesis level after TBI. Briefly, brain sections were incubated for 12 hours at 4°C with primary antibodies: anti-BrdU sheep polyclonal antibody (1 : 200, GeneTex, GTX21893, Irvine, CA, USA) and anti-NeuN rabbit monoclonal antibody (1 : 100, CST, 24307, Beverly, MA, USA). After being washed three times with PBS, sections were incubated for 1 hour at room temperature with the following secondary antibodies: Alexa Fluor 488-labeled donkey anti-sheep antibody (1 : 1000, Invitrogen, A-11015, Eugene, OR, USA) and Alexa Fluor 594-labeled donkey anti-rabbit IgG antibody (1 : 1000, Invitrogen, R-37117, Eugene, OR, USA). After three times washing with PBS, an antifade mounting medium (Electron Microscopy Sciences, CAT17895-01, Hatfield, PA, USA) was used to mount section before cover slipping. Negative controls were set to verify the immunolabeling specificity.

Images were captured under a confocal laser scanning microscope (FV1000, Olympus, Tokyo, Japan) with a FLUOVIEW image system (v.1.4a, Olympus, Tokyo, Japan), assembled in Photoshop 7.0 software (Adobe Systems, San Jose, CA, USA). BrdU/NeuN double-positive cells at SGZ of five consecutive visual fields (400x) in each section were counted. The average number of positive cells in the five visual fields was viewed as the number of positive cells for each section, and the average number of positive cells in five sections was regard as the final number of newborn neurons in each mouse brain.

### 2.6. Western Blot

Hippocampal tissues were isolated from the brain on ice and stored in −80°C. Samples were homogenized and digested in a homogenizer on ice for 15 minutes with a lysis buffer containing 1% NP-40, 150 mM NaCl, 50 mM Tris (pH = 7.4), 1% Triton X-100, 0.5 mM EDTA, 1 mg/ml aprotinin, 1% deoxycholate, 10 mg/ml leupeptin, and 1 mM phenylmethylsulfonyl fluoride. Lysates were centrifuged at 12,000 rpm for 20 minutes at 4°C, and protein concentration was examined with bicinchoninic acid Protein Assay kit (Beyotime, P0011, Shanghai, China). Equivalent amount of protein (40 *μ*g) was loaded and separated by 10% SDS-polyacrylamide gel electrophoresis and transferred to nitrocellulose membrane 4°C for 50 minutes. Membranes were blocked with 5% nonfat milk solution in tris-buffered saline with 0.1% Triton X-100 (TBST) for 1 hour and then incubated overnight at 4°C with appropriate primary antibodies as below: rabbit anti-mouse TLR4 antibody (1 : 1000, Thermo Fisher, PA5-23125, Rockford, IL, USA), rabbit anti-mouse myeloid differentiation factor 88 (Myd88) antibody (1 : 500, Santa Cruz, sc-17320, Dallas, TX, USA), rabbit anti-mouse TNFR-associated factor (TRAF6) antibody (1 : 1000, Novus Biological, NB100-56179, Littleton, CO, USA), rabbit anti-mouse toll/IL-1 receptor domain-containing adapter-induced interferon-*β* (TRIF) antibody (1 : 1000, Enzo Life Sciences, ALX-215-016, Farmingdale, NY, USA), rabbit anti-mouse TRIF-related adaptor molecule (TRAM) antibody (1 : 1000, OriGene, TA-306163, Rockville, MD, USA), rabbit anti-mouse nuclear factor-*κ*B (NF-*κ*B) p65 antibody (1 : 1000, GeneTex, GTX21893, Irvine, CA, USA), and rabbit anti-*β*-actin antibody (1 : 2000, Proteintech, 20536-1-AP, Rosemont, IL, USA). Following three washes in TBST, the membranes were incubated with the second antibody: horse radish peroxidase- (HRP-) conjugated goat anti-rabbit IgG antibody (1 : 20,000, Cell Signaling Technology, 7074, Boston, MA, USA) for 1 hour at room temperature. Immunoreactivity was detected by WesternBright Enhanced chemiluminescence reagents (K12045-d20, Advansta, Menlo Park, CA, USA), and optical densities of the bands were analyzed by Gel-Pro Analyzer software (version 6.0, Media Cybernetics, Rockville, MD, USA).

### 2.7. Enzyme-Linked Immunosorbent Assay

Hippocampal tissues were isolated from the brain and pulverized in a homogenizer under liquid nitrogen. The lysates were incubated in a lysis buffer containing 150 mM NaCl, 10 mM Tris pH 8.0, 1% Triton X-100, 1 mM EDTA, 1 mM phenylmethylsulfonyl fluoride, and 5 *μ*l/ml of protease inhibitor (Sigma-Aldrich, P8340, St. Louis, MO, USA) for 1 hour at 4°C and then centrifugalized at 3000 rpm for 20 minutes. Supernatants were collected for TNF-*α*, IL-1*β*, and IL-6 level measurement with standard ELISA kits (R&D, Minneapolis, MN, USA). The whole experiments were performed under the manufacturer's instructions. Absorbance of samples was measured in a microplate reader, and data was determined in accordance with the standard provided in the kits.

### 2.8. Neurological Severity Score

At 21, 28, and 35 days posttrauma, NSS test was performed to assess the neurobehavioral status of mice by an investigator in a blinded manner. As previously described, the NSS consisted of 10 individual parameters for alertness measurement, balancing examine, and motor ability evaluation [33, 34]. Mouse was awarded one score point for the lack of a tested reflex or the failure to complete a task. The accumulated scores increased with the severity of neurobehavioral deficit. The total score was graded on a scale of 0 to 10, with 0 suggesting a normal behavior status and 10 indicating the maximal neurobehavioral dysfunction.

### 2.9. Water Maze Test

At 31–35 days after TBI, WMT was used to assess neurocognitive function of mice with a 160 cm diameter and 50 cm depth circular tank with a black inner wall filled with water (30 cm depth and 25°C). In accordance with previous studies [35, 36], hidden platform trials were performed to evaluate the learning ability and probe trials were conducted to measure the memory function of mice. For hidden platform trial, the tank was divided into four equal quadrants and a 12 cm diameter black circular platform was hidden 2 cm under water surface in the center of one quadrant. From 31 days posttrauma, each mouse performed four hidden platform trials per day for four days. Briefly, each mouse was allowed to swim freely in the maze and had a maximum of 120 seconds to find the platform. The mouse that failed to reach the platform within 120 seconds was taken out of water and remained on it for 30 seconds. Escape latency referred to the interval between animals was placed into the water and reached the platform. At 35 days posttrauma, hidden platform was removed from the quadrant for probe test. Each mouse was placed into the water to swim freely to find the removed platform. Mouse's trace in quadrant at which the platform was previously located was considered as the route in target quadrant. The times that a mouse swam over the previous platform location were viewed as its platform crossing times. All the parameters in above trails were recorded by a tracking system (DigBeh-MR, Shanghai Auspicious Software Technology Company Limited, China), and the average data was used to analyze the mouse neurocognitive function in different groups.

### 2.10. Statistical Analysis

Data were expressed as means ± SD. One-way analysis of variance (ANOVA) and Tukey HSD post hoc test were applied to analyze statistical significance in GraphPad Prism v.5.0 (GraphPad software, San Diego, CA, USA). Difference of *P* < 0.05 was considered statistically significant.

## 3. Results

### 3.1. Physiological Parameters

Body temperature (BT), heart rate (HR), mean arterial blood pressure (BP), plasma glucose (PG), and arterial blood gas analysis (pH, PaO_2_ and PaCO_2_) of mice were, respectively, determined during the experimental period. No statistical differences of these physiological parameters among groups were observed (*P* > 0.05) (Table 1).

### 3.2. EA Treatment Enhanced Hippocampal Neurogenesis after TBI

**Figure 1:**
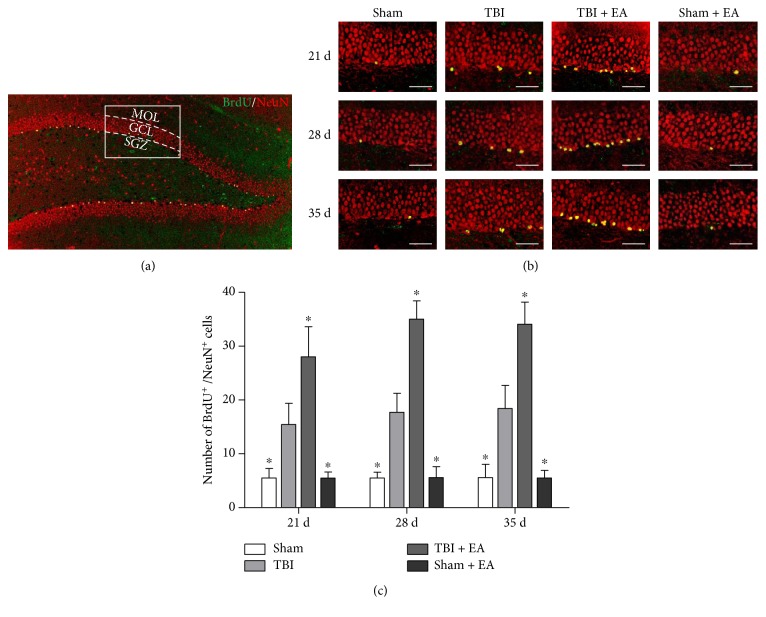
Electroacupuncture (EA) treatment enhanced TBI-induced neurogenesis in the hippocampus after traumatic brain injury (TBI). (a) Coronal section of hippocampal dentate gyrus (DG), stained with BrdU (green fluorescence)/NeuN (red fluorescence). MOL, GCL, and SGZ referred, respectively, to molecular layer, granular cell layer, and subgranular zone in DG. The white pane representing one visual field under confocal laser scanning microscope was shown in (b). (b) Representative immunofluorescence (IF) microphotographs of SGZ in the sham, TBI, TBI + EA, and sham + EA groups (*n* = 9 in each group) at 21, 28, and 35 days postinjury. The newly generated neurons were double labeled with BrdU/NeuN and merged into yellow. (c) Quantitation analysis revealed that, compared with the sham group, the number of BrdU/NeuN-positive cells was notably increased in the TBI group and EA treatment induced much more double-positive cells in the TBI + EA group than in the TBI and sham + EA groups at the three examined time points. Scale bar: 50 *μ*m. ^∗^*P* < 0.05 versus the TBI group.

As shown in Figure 1, the newborn neurons in the hippocampus were double labeled with BrdU (green fluorescence)/NeuN (red fluorescence), which were mainly located in the SGZ of DG. Compared with the sham group, the number of BrdU/NeuN-positive cells in the TBI group was increased at 21, 28, and 35 days posttrauma (*P* < 0.05). Importantly, there were more double-labeled cells in SGZ of the TBI + EA group than the TBI and sham + EA groups (*P* < 0.05), indicating that EA treatment further enhanced the TBI-induced neurogenesis in the hippocampus after TBI. Additionally, the data showed no significant change of BrdU/NeuN-positive cells between the sham group and the sham + EA group.

### 3.3. EA Treatment Inhibited the Expression of TLR4 in the Hippocampus after TBI

**Figure 2:**
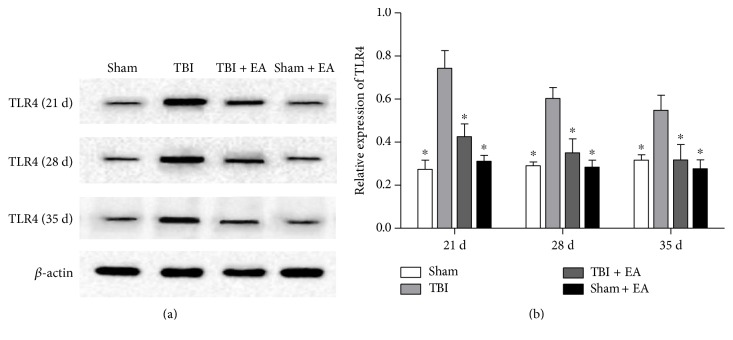
EA treatment inhibited the level of toll-like receptor 4 (TLR4) in the hippocampus after TBI. (a) Representative Western blot (WB) bands of hippocampal TLR4 expression in the sham, TBI, TBI + EA, and sham + EA groups (*n* = 9 in each group) at 21, 28, and 35 days posttrauma. (b) Quantitative analysis suggested that hippocampal TLR4 protein significantly increased in the TBI group compared with the sham group. And EA treatment caused an evident suppression of TBI-induced TLR4 upregulation in the TBI + EA group compared with the TBI and sham + EA groups. ^∗^*P* < 0.05 versus the TBI group.

As shown in Figure 2, the TLR4 protein was increased in the TBI group compared to the sham group (*P* < 0.05), and the TLR4 level in the TBI + EA group was significantly decreased compared with that in the TBI and sham + EA groups, respectively, at 21, 28, and 35 days posttrauma (*P* < 0.05). However, there was no statistical difference of TLR4 expression between the sham and sham + EA groups. These results indicated that EA treatment caused an inhibition of TLR4 expression in the hippocampus after TBI.

### 3.4. Activation of TLR4 Blocked the Enhancement of Hippocampal Neurogenesis Induced by EA Treatment after TBI

**Figure 3:**
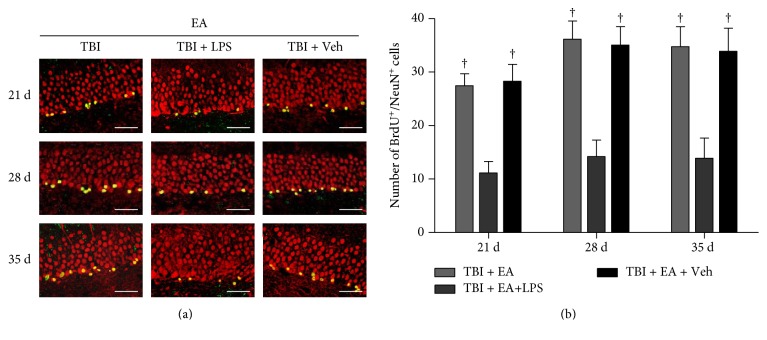
Lipopolysaccharide (LPS) was administrated in lateral ventricle of the brain to activate TLR4 signaling pathway. TLR4 activation attenuated the EA-induced neurogenesis in SGZ of the hippocampus after TBI. (a) Representative BrdU/NeuN double-labeled microphotographs of SGZ in TBI + EA, TBI + EA + LPS, and TBI + EA + Veh groups (*n* = 9 in each group) at 21, 28, and 35 days posttrauma. (b) Statistical data showed that, compared with TBI + EA group, BrdU/NeuN positive cells were significantly decreased in TBI + EA + LPS group and maintained at the same level in TBI + EA + Veh group at the three examined time points. Scale bar: 50 *μ*m. ^**†**^*P* < 0.05 versus the TBI + EA + LPS group.

Then, the effect of TLR4 activation on EA-induced hippocampal neurogenesis was evaluated. As shown in Figure 3, LPS administration significantly decreased the number of BrdU/NeuN-positive cells in the TBI + EA + LPS group compared with the TBI + EA group at 21, 28, and 35 days posttrauma (*P* < 0.05), while there was no significant difference between the TBI + EA + Veh group and the TBI + EA group (*P* > 0.05). According to the data of this section and the results of above sections (Sections 3.2 and 3.3), it can be seen that activation of TLR4 abolished the favorable effect of EA on hippocampal neurogenesis posttrauma.

### 3.5. Activation of TLR4 Eliminated the Improvement of Neurocognitive and Neurobehavioral Recovery Elicited by EA Treatment after TBI

**Figure 4:**
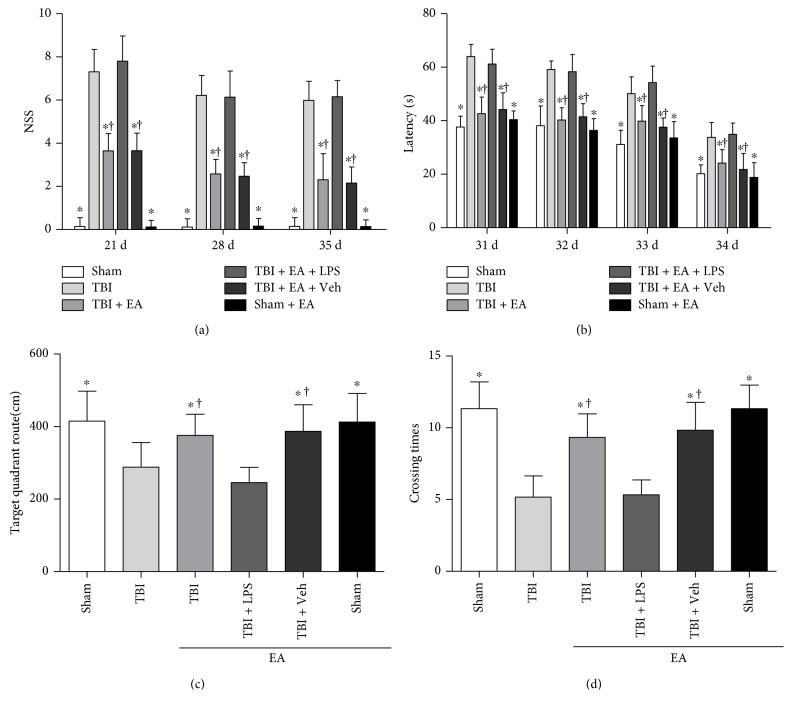
Activation of TLR4 eliminated the promotion of neurological functional recovery induced by EA treatment after TBI. Neurobehavioral and neurocognitive functions of mice in the sham, TBI, TBI + EA, TBI + EA + LPS, TBI + EA + Veh, and sham + EA groups (*n* = 6 in each group) were evaluated by neurological severity score (NSS) and water maze test (WMT). (a) Statistical analysis showed that the TBI group presented higher NSS than the sham group and EA treatment gave rise to an obvious reduction of the severity score in the TBI + EA group. However, LPS administration in TBI + EA + LPS group induced much poorer performance of mice in NSS test compared with the TBI + EA group. (b) Compared with the sham group, mice in the TBI group spent longer escape latency to find the hidden platform at 31–35 days after TBI. EA treatment markedly shortened the latency in the TBI + EA treatment, but the effect was abolished by administration of LPS in the TBI + EA + LPS group. (c, d) The TBI group exhibited less target quadrant route and shorter platform crossing times than the sham group at 35 days posttrauma. EA treatment remarkably increased the route and crossing times of the TBI + EA group, but the favorable role was eliminated by LPS-induced TLR4 activation in the TBI + EA + LPS group. Nevertheless, difference of the above four indexes between the TBI + EA + Veh and TBI + EA groups did not reach the statistically significant level (*P* > 0.05). ^∗^*P* < 0.05 versus the TBI group, ^**†**^*P* < 0.05 versus the TBI + EA + LPS group.

WMT and NSS were conducted to investigate the effect of TLR4 activation on neurocognitive and neurobehavioral functions induced by EA treatment (Figure 4). In cognition assessment, mice in the TBI group spent longer escape latency to find the hidden platform the than sham group, but the mice in the TBI + EA group exhibited shorter latency compared with the TBI and sham + EA groups (*P* < 0.05). Platform crossing times and target quadrant route of the TBI group were less than those of the sham group, but the two indexes were significantly increased in the TBI + EA group compared with the TBI and sham + EA groups (*P* < 0.05). In neurological evaluation, severity score of the TBI group was higher than those of the sham group, but a notable improvement was observed in the TBI + EA group compared with the TBI and sham + EA groups (*P* < 0.05). These results showed that EA treatment rescued the neurocognitive and neurobehavioral deficits caused by TBI.

Compared with the TBI + EA group, LPS administration in the TBI + EA + LPS group caused much poorer performance of mice in neurocognitive and neurobehavioral tests, such as longer escape latency, less crossing times and route, and higher severity scores (*P* < 0.05). Additionally, differences of the above four indexes between the TBI + EA + Veh and TBI + EA groups, as well as between the sham and sham + EA groups, did not reach the statistically significant level (*P* > 0.05). Taken together, it can be inferred that LPS induced TLR4 activation and eliminated the protective effect of EA on neurocognitive and neurobehavioral functions posttrauma.

### 3.6. EA Treatment Suppressed the Activity of TLR4 Downstream Cascade in the Hippocampus following TBI

After understanding the association between EA treatment and TLR4 in hippocampal neurogenesis, we further examined how the activity of TLR4 downstream signaling pathway in response to EA treatment at 35 days after TBI. WB was performed to determine the level of Myd88 and TRAF6 in TLR4/Myd88-dependent pathway; TRIF and TRAM in TLR4/TRIF-dependent pathway; as well as the two pathways' common target molecule NF-*κ*B.

**Figure 5:**
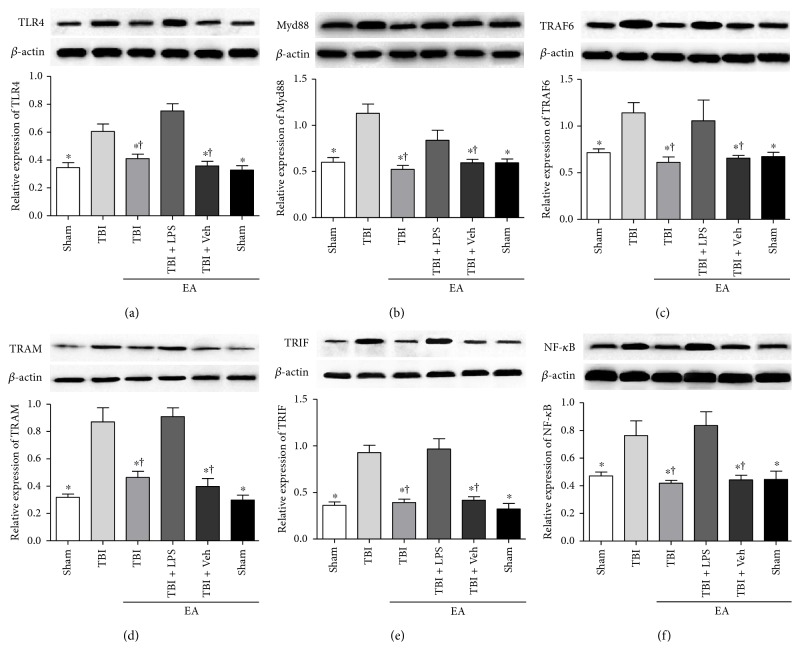
EA treatment exerted an inhibitory effect on both of the two downstream pathways of TLR4 in the hippocampus posttrauma. Hippocampal TLR4, Myd88, TRAF6, TRAM, TRIF, and NF-*κ*B expression of the sham, TBI, TBI + EA, TBI + EA + LPS, TBI + EA + Veh, and sham + EA groups (*n* = 3 in each group) were detected by WB at 35 days after TBI. (a–f) The six kinds of proteins in TLR4/Myd88*κ*B and TLR4/TRIF/*κ*B signaling pathways were upregualted in the TBI group compared with the sham group and the sham + EA group. EA treatment resulted in a significant decrease in the TBI + EA group, but the effects were attenuated by LPS administration in the TBI + EA + LPS group. No difference of the proteins between the TBI + EA and TBI + EA + Veh groups was observed. ^∗^*P* < 0.05 versus the TBI group, ^**†**^*P* < 0.05 versus the TBI + EA + LPS group.

As shown in Figure 5, the expression of TLR4, Myd88, TRAF6, TRAM, TRIF, and NF-*κ*B p65 significantly increased in the TBI group compared to the sham group (*P* < 0.05). EA treatment in the TBI + EA group produced an evident decrease of the six molecule levels in comparison with the TBI and sham + EA groups, but the effect was relieved by administration of LPS in the TBI + EA + LPS group (*P* < 0.05). The difference between the sham and sham + EA group has no statistical significance. These data indicated that TLR4/Myd88/NF-*κ*B and TLR4/TRIF/NF-*κ*B axles were activated posttrauma, and EA treatment exerted a restraining influence on both of the two pathways.

### 3.7. EA Treatment Alleviated the Expression of TLR4 Cascade-Induced Inflammatory Cytokines in the Hippocampus after TBI

**Figure 6:**
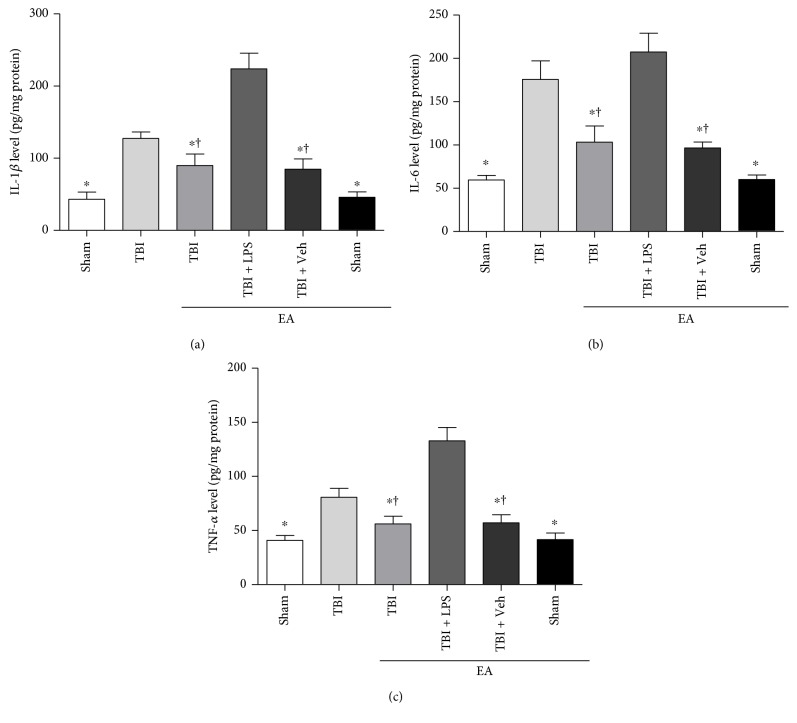
EA treatment suppressed the level of inflammatory cytokines in the downstream of TLR4 signaling pathway posttrauma. TNF-*α*, IL-1*β*, and IL-6 expression in the hippocampus were determined by ELISA. (a–c) Quantitative analysis showed that three inflammatory cytokines were elevated in the TBI group compared with the sham and sham + EA groups. EA treatment induced significant abatement of these cytokines in the TBI + EA treatment. Reversely, LPS administration abrogated the inhibitory effect of EA on TLR4-mediated inflammation in the TBI + EA + LPS group. There was no significant difference in these inflammatory cytokine expression between the TBI + EA + Veh and TBI + EA groups. ^∗^*P* < 0.05 versus the TBI group, ^**†**^*P* < 0.05 versus the TBI + EA + LPS group.

We further investigated the effect of EA treatment on inflammatory cytokine expression in the downstream of TLR4/Myd88/NF-*κ*B and TLR4/TRIF/NF-*κ*B axles at 35 days after TBI. ELISA was performed to determine the level of inflammatory cytokines including TNF-*α*, IL-1*β*, and IL-6 in the hippocampus (Figure 6). Comparison with the sham and sham + EA groups showed that the level of these inflammatory cytokines was markedly elevated in the TBI group but was significantly decreased in the TBI + EA group (*P* < 0.05). However, administration of LPS in the TBI + EA + LPS group induced an evident increase of the inflammatory cytokines in comparison with the TBI + EA group (*P* < 0.05). Significant difference of cytokine expression between the TBI + EA + Veh and TBI + EA groups, as well as between the sham and sham + EA groups, was not observed (*P* > 0.05). Taken together, the data suggested that brain trauma triggered the inflammatory response mediated by TLR4 cascade in the hippocampus, and EA treatment mitigated the inflammation through downregulation of inflammatory cytokines, which might be helpful to posttraumatic neurogenesis.

## 4. Discussion

In the present study, these above three parts of experiments were performed with a purpose to investigate the effect and potential mechanisms of EA treatment on hippocampal neurogenesis following TBI. It was explored in the first part that how neurogenesis and TLR4 expression in the hippocampus responded to EA intervention in experimental TBI mice. Second, the role of TLR4 in the EA-induced neurogenesis and functional recovery following TBI was investigated in order to discover the potential mechanism of EA in inducing posttraumatic neurogenesis. Last, the influence of EA treatment upon TLR4 downstream pathways and inflammatory cytokines was examined. The corresponding results suggested that EA treatment produced a beneficial effect on neurogenesis and an inhibitory action on the level of TLR4 in the hippocampus after TBI. TLR4 activation induced by LPS eliminated the promotion of EA on hippocampal neurogenesis and neurological functions. In addition, it is interesting to note that EA treatment caused a repression on the activity of TLR4/Myd88- and TLR4/TRIF-dependent pathways, as well as the expression of downstream inflammatory cytokines posttrauma.

As one of the most studied complementary and alternative medicines, acupuncture has been applied to treat pathological conditions of the CNS since thousands of years ago in China [6, 9]. Increasing studies suggest that the easily operated and economical EA is a promising therapy for hypoxic-ischemic brain injury. Pretreatment or treatment of EA worked against cerebral ischemia and reperfusion damage through regulation of neuronal excitotoxicity, apoptosis, oxidative stress, and inflammation in ischemic stroke [37–39]. It had also been shown that EA took definite benefits on the improvement of neural repairing after stroke [40–42]. In addition, the study by Chuang et al. stated that EA intervention at the acute stage of TBI resulted in a decrease of transforming growth-interacting factor in injured area and an increase of the regional cerebral blood flow in rats, which contributed to reduce neuronal apoptosis and improve neurological outcomes posttrauma [43]. Zhou and his colleagues reported that EA at ST36 acupoint improved neurological function recovery by upregulating angiopoietin 1 and 2 expression in the injured cortex of cerebral hemorrhagic rat [12]. However, little is known about the efficacy of EA in neural rehabilitation and functional recovery following TBI. In the current study, we found that EA stimulation at ST36 and GV40 acupoints produced a beneficial effect on hippocampal neurogenesis at 7, 21, and 35 days posttrauma. Although Wong et al. have tried to assess the efficacy and safety of acupuncture for TBI patients in a series of systematic reviews published from 2011 to 2013 [44–46], they could not draw any conclusion because of the insufficient clinical random controlled trials and low methodological quality. In some extent, this finding is controversial with the above experimental findings, suggesting that further large amount and well-designed studies are required to elucidate the exact role of EA in clinical TBI patients.

It has been documented that various molecules and signals are involved in the protective effect of EA on neurological disorders although the precise mechanism is not yet definitely elaborated. Several previous studies suggested that TLR4 might be implicated in the neuroprotective effect of EA in cerebral ischemic injury tolerance [13, 47]. In the present study, the possible involvement of TLR4 in EA-induced hippocampal neurogenesis was investigated. It was observed that both protein and mRNA level of TLR4 in the hippocampus were augmented at 7, 21, and 35 days after TBI, which consisted with the data in our previous study [27]. Importantly, EA treatment reversed the increase of hippocampal TLR4 expression posttrauma. TLR4 activation blocked the promotion of NSC neuronal differentiation elicited by EA treatment in SGZ. Similarly, EA stimulation improved neurological deficits and spatial learning and memory function, which was closely associated with neural repairing posttrauma. But the favorable effects were abrogated by LPS-induced TLR4 activation. Therefore, it could be inferred that the inhibition of TLR4 might be an essential mediator for the effect of EA on hippocampal neurogenesis after TBI.

TLR4 has been identified to play a fundamental role in the regulation of innate immune response and adaptive immune system via TLR4/Myd88- and TLR4/TRIF-dependent pathways [48]. Previous studies showed that the level of TLR4 downstream adaptor protein was upregulated in traumatic brain and inhibition of Myd88 could remarkably improve neuronal survival and neurological function [49, 50]. In addition, preinjury antagonism of TLR4 gave rise to a notable decrease of MyD88 and TRIF expression in the hippocampus of a TBI rat model, which might be related to the neurocognitive function posttrauma [51]. Recently, quite a few studies discovered that TLR4 and its downstream signaling molecules were responsible for the modulation of adult hippocampal neurogenesis and neural plasticity [24, 52, 53]. Our present work revealed that EA treatment possessed an inhibitory effect on the upregulation of TLR4 downstream signaling molecules posttrauma, including Myd88 and TRAF6 in TLR4/Myd88-dependent pathway and TRAM and TRIF in TLR4/TRIF-dependent pathway. As noted previously, NF-*κ*B was one of the most important effectors in the signaling transduction of both Myd88- and TRIF-dependent pathways [16, 54]. Accordingly, we analyzed whether the NF-*κ*B expression in the hippocampus responds to EA treatment after TBI. Our results showed that the TBI-induced hippocampal NF-*κ*B elevation was significantly inhibited by EA treatment, which varied in the similar pattern to Myd88, TRAF6, TRAM, and TRIF. Taken together, these results indicated that EA treatment could repress the activities of both TLR4/Myd88- and TLR4/TRIF-dependent pathways in the hippocampus following TBI, and this may be one of the mechanisms by which EA inhibits posttraumatic NF-*κ*B expression.

It is well known that neuroinflammation, one of the prominent pathological responses in injured brain, is a double-edged sword for neural plasticity depending on the balance between neurotoxic and neuroprotective effect in the hippocampus [55–57]. On the one hand, inflammatory response is propitious to the initiation of NSC proliferation and differentiation in pathological status of the CNS [58, 59]; on the other hand, overproduced inflammatory cytokines in neurogenic niche are detrimental to NSC survival and fate [25, 60, 61]. Studies have noted that TLR4-mediated inflammation is critical for the property of endogenous NSCs during the process of neurogenesis [23, 62]. Myd88- and TRIF-dependent pathways have been identified as contributing factors for activating NF-*κ*B to trigger the release of inflammatory cytokine and chemokine in TLR4 downstream cascade [63–65]. In the present experiment, it was observed that the level of TNF-*α*, IL-1*β*, and IL-6 in the hippocampus was amplified at 35 days after TBI. EA treatment led to an evident downregulation of these proinflammatory cytokines, and this efficacy was eliminated by LPS administration. In view of previous findings and our present results, it can be speculated that, under the given parameters of this study, EA improved endogenous neurogenesis depending on its inhibition of TLR4/Myd88/NF-*κ*B and TLR4/TRIF/NF-*κ*B axle-induced inflammatory cytokines in the hippocampus after TBI. Although we cloud not exclude the possibility that this conclusion may be much associated with the selected time window and ways of EA intervention on the employed TBI model in the present study, our obtained results are consistent with previous literatures which described the efficacy of EA treatment in other neurological disorders, including ischemia stroke and surgical traumatic stress [47, 66].

Indeed, there are some limitations in this study since the experimental design was *in vivo*. It is hard to illustrate that the posttraumatic expression of TLR4 downstream mediators and inflammatory cytokines results from which type of cells in the neurogenesis niche. Therefore, further *in vitro* study is required to recognize the exact role of TLR4 signaling pathway in NSC proliferation and differentiation, as well as in the activation of hippocampal microglia and astrocyte after TBI. In addition, it is beyond the scope of this study to differentiate individual or synergistic effects of the inflammatory cytokines on hippocampal endogenous neurogenesis under the treatment of EA *in vivo*. Thus, such analysis needs to be performed in future *in vitro* investigation.

## 5. Conclusion

In summary, it is observed for the first time that EA treatment promoted hippocampal neurogenesis and neurological function recovery in TBI mice, at least in part, by suppressing TLR4 signaling pathway and its downstream proinflammatory response. Although preliminary, this present study paved a valuable way for further study and indicated that EA intervention might be one of the promising strategies to improve neurogenesis and neurological function restoration after TBI.

## Figures and Tables

**Table 1:** Animal physiological parameters.

Group	BT (°C)	HR (/min)	BP (mmHg)	PG (mmol/l)	PaO_2_ (mmHg)	PaCO_2_ (mmHg)	pH
Sham	37.6 ± 1.3	372.2 ± 28.5	133.4 ± 9.7	7.6 ± 0.3	97.4 ± 10.1	42.0 ± 5.9	7.37 ± 0.03
TBI	37.3 ± 1.6	369.5 ± 17.4	126.7 ± 14.4	7.0 ± 0.4	98.9 ± 13.5	39.3 ± 4.2	7.40 ± 0.06
TBI + EA	37.0 ± 1.9	365.3 ± 29.9	134.6 ± 11.3	7.4 ± 0.6	95.4 ± 17.8	43.6 ± 5.4	7.42 ± 0.03
TBI + EA + LPS	36.7 ± 1.1	373.5 ± 22.3	130.8 ± 14.9	7.7 ± 0.4	95.8 ± 13.5	40.5 ± 4.9	7.41 ± 0.06
TBI + EA + Veh	37.2 ± 0.7	371.9 ± 15.2	134.1 ± 7.2	7.4 ± 0.2	96.6 ± 10.8	44.7 ± 6.5	7.36 ± 0.04
Sham + EA	36.9 ± 1.5	370.4 ± 13.8	131.6 ± 10.2	7.5 ± 0.9	96.5 ± 9.2	44.2 ± 4.1	7.33 ± 0.02

Data are expressed as mean ± SD and no statistical difference between all groups.
